# Evaluation of the Antimicrobial Activity of the Decoction of *Tropidurus hispidus* (Spix, 1825) and *Tropidurus semitaeniatus* (Spix, 1825) Used by the Traditional Medicine

**DOI:** 10.1155/2012/747969

**Published:** 2011-06-30

**Authors:** Israel J. M. Santos, Edinardo F. F. Matias, Karla K. A. Santos, Maria F. B. M. Braga, Jacqueline C. Andrade, Teógenes M. Souza, Francisco A. V. Santos, Ana Carla A. Sousa, José G. M. Costa, Irwin R. A. Menezes, Rômulo R. N. Alves, Waltecio O. Almeida, Henrique D. M. Coutinho

**Affiliations:** ^1^Laboratory of Zoology, Regional University of Cariri-URCA, Pimenta 63105-000, Crato, CE, Brazil; ^2^Laboratory of Microbiology and Molecular Biology, Regional University of Cariri-URCA, Pimenta 63105-000, Crato, CE, Brazil; ^3^Laboratory of Natural Products Research, Regional University of Cariri-URCA, Pimenta 63105-000, Crato, CE, Brazil; ^4^Laboratory of Phamacology and Medicinal Chemistry, Regional University of Cariri-URCA, Pimenta 63105-000, Crato, CE, Brazil; ^5^Department of Biology, Paraiba State University-UEPB, Joăo Pessoa 58429-500, PB, Brazil

## Abstract

*Tropidurus hispidus* and *Tropidurus semitaeniatus* are two lizard species utilized in traditional medicine in Northeast Brazil. Their medicinal use includes diseases related with bacterial infections such as tonsillitis and pharyngitis. They are used in the form of teas (decoctions) for the treatment of illnesses. In this work, we evaluated the antimicrobial activity of the decoctions of *T. hispidus* (DTH) and *T. semitaeniatus* (DTS) against bacterial strains, namely, standard and multiresistant *Escherichia coli*, *Staphylococus aureus*, and *Pseudomonas aureuginosa*, alone and in combination with aminoglycoside antibiotics. The decoctions were prepared using the whole body of the dried lizards, and the filtrate was frozen and lyophilized. When tested alone, the samples did not demonstrate any substantial inhibition of bacterial growth. However, in combination with antibiotics as aminoglycosides, decoctions reduced the minimal inhibitory concentration (MIC) of the assayed antibiotics against multiresistant strains of *S. aureus* and *P. aureuginosa*. Chemical prospecting tests revealed the presence of alkaloids in DTS. This is the first study evaluating the medicinal efficacy of *T. hispidus* and *T. semitaeniatus* and contributes to the list of new sources of medicines from natural products of animal origin.

## 1. Introduction

Natural substances from animals, plants, and minerals have provided a continuous source of medications [1]. In Brazil, as in other countries, animals and plants have been widely utilized since antiquity by the traditional medicine [2, 3], and according to Alves and Rosa [4], they have played a significant role in the healing arts up to nowadays. 

Despite their prevalence in the practice of traditional medicine throughout the world, the medicinal use of animals have often been neglected in research, compared to medicinal plants [5]. According to Alves et al. [6], emphasis has been placed for the most part on plant-based medications more than on those from animal origin. Besides, plants are easier to collect, store, and sell. However, recent publications have demonstrated the importance of zootherapy in different sociocultural environments worldwide, and examples of the use of remedies derived from animals can currently be found in many urban and semiurban localities, particularly in developing countries [2, 3, 7, 8].

Reptiles are among the species most utilized in popular medicine, and their role in practices and beliefs related to the treatment and/or prevention of diseases has been reported by different traditional communities worldwide [4, 7–11]. Despite the extensive use of reptiles for medicinal purposes, there is a general lack of detailed information about the exploitation of these animals and their impact on the species involved [11].

Among the species utilized in traditional medicine in Brazil, we can cite *Tropidurus hispidus *and* T. semitaeniatus. Tropidurus semitaeniatus* (Spix, 1825) is endemic to the “Caatinga” biome. Popularly known as the “outcrop lizard,” it is a small lizard with a diurnal habit. It is found on broad rocky surfaces (outcrops), and with a dorsoventral flattened body, specialized at getting into small cracks in the rocks, where it is protected and probably remains during the warmest hours of the day [12]. *T. semitaeniatus* is a carnivorous animal, with a sit-and-wait feed strategy, consuming a large variety of preys, mainly ants [13]. In popular medicine, *T. semitaeniatus* is indicated for the treatment of measles, asthma, alcoholism, dermatomycosis, and chickenpox [6].

*Tropidurus hispidus *(Spix, 1825), also known as the “lava lizard” or “catenga,” inhabits the Brazilian Northeastern region [14, 15]. It is found in diverse habitats, mainly on tree trunks, rocks, and walls [14, 15] and lives mainly in open areas. This is a diurnal and territorial lizard with a sedentary-opportunistic feed strategy [13, 15–18]. It feeds mainly on arthropods, some plants, flowers, and small vertebrates [14, 19]. According to Barbosa [20], *T. hispidus* is used in the treatment of chickenpox in a community in Paraiba State. Alves and Rosa [3] noted in their studies the popular use of this species in the treatment of sore throat, tonsillitis, and pharyngitis. 

According to Freire [21] and Marques [22], the utilization of these lizards in traditional medicine is also associated with the treatment of inflammation, dermatitis, venereal diseases, and snake bites, being consumed in the form of a decoction, and because of the small size of the specimens, it is used whole in the preparation of a tea. Alves et al. [6] also described other forms of use for these species in which a tea (decoction) is included, besides the ingestion of a broth of cooked meat and the application of the live animal on the affected area. Many of these diseases such as inflammation and dermatitis can be associated with pathogenic microorganisms, including bacteria and fungi, which suggest a possible antimicrobial potential for these species. 

Traditional medicine, in general, represents a field in which there is still little research in terms of evaluation of therapeutic or clinical potential [23], and few studies have been done until now to demonstrate the clinical efficacy of animal products for medicinal purposes [24]. Therefore, the aim of this work was to determine the antimicrobial activity of decoctions prepared with the lizards *T. hispidus* and *T. semitaeniatus*, tested alone and in combination with antibiotics.

## 2. Material and Methods

### 2.1. Zoological Material

The animals were collected in the municipality of Crato (7°14′03′′S × 39°24′34′′W), Ceará, Brazil in April 2010. They were caught manually and with air pistols by rummaging through habitats where these animals can be found (Permission for collection: 154/2007 no. 23544-1 process no. 17842812). Once the lizards were collected and sacrificed, their skins were removed and dried in a drying oven to prepare extracts. Control specimens were fixed in 70% alcohol and deposited in the zoology collection of the Universidade Regional do Cariri/LAZ-URCA (Table 1).

### 2.2. Preparation of Decoctions of *T. hispidus* (DTH) and *T. semitaeniatus* (DTS)

The decoctions of *T. hispidus *and* T. semitaeniatus* were prepared by submersing the whole lizards, already oven-dried, in boiling distilled water for 2 h. Afterward, the decoction was filtered, frozen, and later lyophilized. A concentrated form was used in the antimicrobial assays. The yields for the decoctions are shown in Table 2. The decoctions were then stored in a freezer for future analyses.

### 2.3. Strains

The experiments were carried out using the following bacteria: clinical isolates of *Escherichia coli* (EC27), *Staphylococcus aureus* 358 (SA358), and *Pseudomonas aureuginosa* PA RB1 and the standard strains *E. coli *ATCC 10536, *S. aureus* ATCC 25923, and *P. aeruginosa* ATCC 15692 [25]. All the strains were maintained on heart infusion agar slants (HIA, Difco), and, before the assays, the cells were grown overnight at 37°C in brain heart infusion broth (BHI, Difco).

### 2.4. Drugs

The antibiotics utilized, gentamicin, kanamycin, amikacin, and neomycin, were obtained from Sigma Chemical Corp, St. Louis, Mo, USA. All the drugswere dissolved in sterile water before use.

### 2.5. Drug Susceptibility Tests

The test solution of the decoctions of the two species was prepared by dissolving 10 mg of the samples in 1 mL of dimethylsulfoxide (DMSO-Merck, Darmstadt, Germany), obtaining an initial concentration of 10 mg/mL. This solution was then diluted to 1024 *μ*g/mL using sterile water. The minimal inhibitory concentrations (MIC) of the extracts were determined using microdilution assays in BHI broth with bacterial suspensions of 10^5^ CFU/mL and drug concentrations varying from 1024 to 1 *μ*g/mL (in 2-fold serial dilutions) [26]. MIC was defined as the lowest concentration of drug at which no bacterial growth was observed. For the evaluation of extracts for antibiotic-modifying activity, MICs of the antibiotics were determined in the presence and absence of each decoction at subinhibitory concentrations (128 *μ*g/mL), and the plates were incubated for 24 h at 37°C.

### 2.6. Chemical Prospecting

The chemical tests to determine the presence of heterosides, saponins, tannins, flavonoids, steroids, triterpenes, cumarins, quinones, organic acids, and alkaloids were performed according to the method described by Matos [27]. The tests are based on visual inspection for a color changes or formation of a precipitate after the addition of specific reagents. The results obtained are presented in Table 3.

## 3. Results

The decoctions of the lizards *T. hispidus* and *T. semitaeniatus* did not show a clinically relevant antibacterial activity, presenting a MIC ≥1024 *μ*g/mL against all bacterial strains tested, suggesting that these lizards are ineffective in traditional medicine against bacterial infections. DTH and DTS were tested for possible antibacterial activity when combined with commonly used antibiotics. No effect of any decoction was observed against the multiresistant strain of *E. coli*-EC27. Against the strain SA358, DTS combinated with a kanamycin and amikacin significantly reduced the MIC of these antibiotics as observed in Table 4. DTH also enhanced the action of the kanamycin against the same bacterial strain. Against the multiresistant clinical isolate and *Pseudomonas aureuginosa *RB1, both DTH and DTS showed synergism with the aminoglycosides neomycin and gentamicin (Table 4). The chemical prospecting tests demonstrated the presence of alkaloids in the decoction of *T. semitaeniatus*, but not in the case of *T. hispidus,* as seen in Table 3. 

## 4. Discussion

The present study demonstrated that decoctions of *T. hispidus* and *T. semitaeniatus* did not presents clinically relevant antibacterial activity, with MIC of ≥1024 *μ*g/mL against all the strains used. Similar results were obtained in a study by Ferreira et al. [28], demonstrating the lack of *in vitro* antimicrobial activity of body fat from *Tupinambis merianae*, which is used in popular medicine against bacterial infections caused by *E. coli* and *S. aureus*, besides, several proteins and peptides from animals present antibacterial activity [29].

On the other hand, a synergistic effect was observed between the extracts with aminoglycosides, reducing the MIC of the antibiotics was also observed in other studies with natural products isolated from animals and plants [30–32]. Therefore, there is a need to understand how these substances act in order to increase the activity of conventional antibiotics, since a substantial decrease in the concentration of aminoglycosides would be a promising improvement in the chemotherapy of infections. According to Matias et al. [33], several components of the extracts can act as cell permeabilizers, increasing the cellular uptake of antibiotics [34]. Interference with bacterial enzyme systems can also be a potential mechanism of action [35]. These mechanisms of action can be involved in the combination of an antibiotic with a natural product at a subinhibitory concentration [36, 37]. 

The presence of alkaloids in the decoction of *T. semitaeniatus* used in these antimicrobial assays can be a strong indication that these substances present the antibiotic-modifying activity, since these extracts potentiated the antibiotic action (Table 4). Studies have demonstrated different pharmacological activities of alkaloids [38, 39]. In the case of DTH, which does not contain alkaloids but still showed synergism with particular aminoglycosides, other possible bioactive substances not detected may be responsible for its synergistic effect, necessitating further studies to identify these natural products.

It is important to note that the use of natural products combined with conventional drugs has been previously described. Calvet-Mir et al. [40] reported the use of traditional medicine products in combination with Western medicine for the treatment of diarrhea, vomiting, and stomach ache. Vandebroek et al. [41] reported on the use of natural products and commercial medications together for the treatment of diseases of the respiratory and digestive tracts.

According to Ferreira et al. [42], studies of substances from reptiles must be stimulated to determine their pharmacological activities. Ciscotto et al. [43] described the antibacterial and antiparasitic activities of l-amino acid oxidase from the venom of this snake. Morais et al. [44] reported the anticoagulant activity of antithrombin factor from *Bothrops jararaca* (Wied, 1924) venom. Products from other species of reptiles were also studied in attempt to elucidate their pharmacological proprieties. Liu et al. [45] demonstrated the antitumor effect of extracts of the lizard *Gecko japonicas *(Schlegel, 1836), which is widely utilized in Chinese traditional medicine. The lysozymes of the turtles *Trionyx sinensis* (Wiegmann, 1835), *Amyda cartilaginea* (Boddaert, 1770), and *Chelonia mydas *(Linnaeus, 1758) demonstrated a strong antibacterial activity [46].

## 5. Conclusion

The decoctions of *T. hispidus* and *T. semitaeniatus*, alone, did not show antimicrobial activity, suggesting the ineffectiveness of products derived from these animals for the treatment of bacterial infectious diseases in traditional medicine. However, the decoctions were found to be effective when combined with aminoglycoside, demonstrating a pharmacological potential to enhance the antibiotic activity. Further studies with natural products of animal origin are needed since this field still remains few explored compared to phytotherapeutic substances, and the medicinal potential of products derived from animals can lead to notable advances in conventional medicine, as well as to the development of management techniques in the conservation of species with potential medicinal use. 

## Figures and Tables

**Table 1:** Species utilized in the antimicrobial analyses.

Species	University/Archive no.	
*Tropidurus hispidus*	Universidade Regional do Carri-URCA	LZ-847
*Tropidurus semitaeniatus*	Universidade Regional do Carri-URCA	LZ-926

**Table 2:** Fresh weight, dry weight, and yield of decoctions of *Tropidurus hispidus *and* Tropidurus semitaeniatus *(g).

Species	Fresh weight	Dry weight	Solvent	Yield of crude extract
*Tropidurus hipidus*				
Whole animal	193.5	52.00	Distilled water (DTH)	11.0539
*Tropidurus semitaeniatus*				
Whole animal	94.68	27.7067	Distilled water (DTS)	4.4885

DTH: decoction of *Tropidurus hispidus; *DTS: decoction of *Tropidurus semitaeniatus*.

**Table 3:** Results of chemical prospecting of decoctions of *Tropidurus hispidus *and* Tropidurus semitaeniatus. *

Classes of secondary metabolite	DTH	DTS
Phenols	−	−
Pyrogallic tannins	−	−
flobatenic tannins	−	−
Anthocyanins	−	−
Anthocyanidines	−	−
Flavones	−	−
Flavonols	−	−
Xanthones	−	−
Chalcones	−	−
Aurones	−	−
Flavanonols	−	−
Leucoanthocyanidins	−	−
Catechins	−	−
Flavanones	−	−
Terpenes	−	−
Alkaloids	−	+

DTH: decoction of *Tropidurus hispidus; *DTS: decoction of *Tropidurus semitaeniatus*. (+) presence and (−) absence.

**Table 4:** MIC values (*μ*g/mL) of aminoglycosides in the absence and presence of 128 *μ*g/mL of decoctions of *T. hispidus *or* T. semitaeniatus *against *Escherichia coli* 27, *Staphylococcus aureus* 358, and *Pseudomonas aeuruginosa *RB1.

	EC 27			SA 358			PA RB1		
	MIC	MIC combined		MIC	MIC combined		MIC	MIC combined	
		DTH	DTS		DTH	DTS		DTH	DTS
Antibiotic									
Kanamycin	78.1	78.1	78.1	156.2	39.1	39.1	625	625	625
Amikacin	78.1	78.1	78.1	156.2	156.2	19.5	312.5	312.5	312.5
Neomycin	78.1	78.1	78.1	78.1	78.1	78.1	625	78.1	156.2
Gentamicin	19.5	19.5	19.5	9.8	9.8	9.8	78.1	19.5	19.5

DTH: decoction of *Tropidurus hispidus; *DTS: decoction of *tropidurus semitaeniatus.*
